# Kindlin-2 Regulates the Oncogenic Activities of Integrins and TGF-β In Triple Negative Breast Cancer Progression and Metastasis

**DOI:** 10.21203/rs.3.rs-3914650/v1

**Published:** 2024-02-05

**Authors:** Neelum Aziz Yousafzai, Lamyae El Khalki, Wei Wang, Justin Szpendyk, Khalid Sossey-Alaoui

**Affiliations:** Case Western Reserve University School of Medicine; Case Western Reserve University School of Medicine; MetroHealth; MetroHealth; Case Western Reserve University School of Medicine

**Keywords:** Kindlin-2, FERMT2, β1-Integrin, ITGB1 TGF-β type 1 receptor, TβRI, Triple Negative Breast Cancer, Tumor progression and metastasis, Hallmarks of Cancer

## Abstract

**Background:**

Kindlin-2, an adaptor protein, is dysregulated in various human cancers, including triple negative breast cancer (TNBC), where it drives tumor progression and metastasis by influencing several cancer hallmarks. One well-established role of Kindlin-2 involves the regulation of integrin signaling, achieved by directly binding to the cytoplasmic tail of the integrin β subunit. In this study, we present novel insights into Kindlin-2’s involvement in stabilizing the β1-Integrin:TGF-β type 1 receptor (TβRI) complexes, acting as a physical bridge that links β1-Integrin to TβRI. The loss of Kindlin-2 results in the degradation of this protein complex, leading to the inhibition of downstream oncogenic pathways.

**Methods:**

Our methodology encompassed a diverse range of in vitro assays, including CRISPR/Cas9 gene editing, cell migration, 3D tumorsphere formation and invasion, solid binding, co-immunoprecipitation, cell adhesion and spreading assays, as well as western blot and flow cytometry analyses, utilizing MDA-MB-231 and 4T1 TNBC cell lines. Additionally, preclinical in vivo mouse models of TNBC tumor progression and metastasis were employed to substantiate our findings.

**Results:**

The investigation revealed that the direct interaction between Kindlin-2 and β1-Integrin is mediated through the C-terminal F3 domain of Kindlin-2, while the interaction between Kindlin-2 and TβRI is facilitated through the F2 domain of Kindlin-2. Disruption of this bridge, achieved via CRISPR/Cas9-mediated knockout of Kindlin-2, led to the degradation of β1-Integrin and TβRI, resulting in the inhibition of oncogenic pathways downstream of both proteins, subsequently hindering tumor growth and metastasis. Treatment of Kindlin-2-deficient cells with the proteasome inhibitor MG-132 restored the expression of both β1-Integrin and TβRI. Furthermore, the rescue of Kindlin-2 expression reinstated their oncogenic activities both in vitro and in vivo.

**Conclusions:**

This study identifies a novel function of Kindlin-2 in stabilizing the β1-Integrin:TβR1 complexes and regulating their downstream oncogenic signaling. The translational implications of these findings are substantial, potentially unveiling new therapeutically targeted pathways crucial for the treatment of TNBC tumors.

## Background

Breast Cancer (BC) ranks as the second leading cause of cancer-related deaths among women in the United States, with nearly 300,000 new cases reported annually and over 43,000 lives lost [1]. The acquisition of metastatic phenotypes accounts for approximately 90% of BC-related deaths [2–4]. Metastatic BC, typically incurable, imposes a median survival of only 1.5 to 3 years for affected patients. Clinically, about 30% of BC patients initially diagnosed with early-stage, noninvasive disease progress to late-stage, metastatic disease, significantly limiting treatment options and resulting in dismal clinical outcomes [2]. This challenge is exacerbated by the heterogeneous nature of BCs, comprising genetically distinct subtypes [5–7], with triple-negative BCs (TNBCs) standing out as particularly lethal due to their highly metastatic behavior and rapid recurrence [8–12]. TNBCs lack expression of hormone receptors (ER-α and PR) and ErbB2/HER2 [8–12], which imposes a hurdle for FDA-approved targeted drug therapies. Additionally, TNBCs often develop resistance to standard-of-care treatments through unidentified mechanisms. Hence, preventing TNBC progression and recurrence emerges as a critical strategy to significantly enhance the clinical course for TNBC patients.

Kindlins, a small gene family of FERM domain-containing adaptor proteins, comprising three members, with Kindlin-2 being the most widely expressed [13, 14]. Dysregulated Kindlin expression is linked to various human pathologies, including cancer [14, 15]. In particular, Kindlin-2 plays a pivotal role in breast cancer progression, promoting metastasis and invasion [16–18]. Kindlin-2 regulates the growth and progression of breast cancer tumors by activating CSF-1-mediated macrophage infiltration, thereby promoting metastatic progression [16]. Its involvement extends to regulating tumor growth and progression by enhancing Wnt signaling through complex formation with β-catenin and TCF4, and contributing to Src-mediated tyrosine phosphorylation of androgen receptor [19][20]. Therefore, Kindlin-2 has been established a major regulator of several hallmarks of cancer [21]. Our group’s studies further established Kindlin-2 as a major driver of the invasion-metastasis cascade in TNBC, influencing epithelial-to-mesenchymal transition (EMT), cancer cell senescence, chemotherapeutic sensitization, and actin-mediated integrin outside-in signaling [13, 22–24]. Noteworthy contributions of Kindlin-2 to breast cancer pathogenicity include the regulation of HIF-1α-mediated activation of tumor angiogenesis, mitotic spindle assembly through inhibiting histone deacetylase 6, maintenance of α-tubulin acetylation, and stabilization of DNA methyltransferase 1 (DNMT1) [25–27]. Both in cancer cells and mammary glands, Kindlin-2 is established as a requirement for BC tumor development and progression in transgenic mice [28]. We also established Kindlin-2’s pivotal role in regulating TNBC progression and metastasis through CSF-1/EGF paracrine signaling and upstream TGF-β [16]. Of particular interest is the interaction between Kindlin-2 and TGF-β Type One Receptor (TβRI), elucidated by Wei and colleagues [29].

Given Kindlin-2’s role as a coactivator of integrin activities and its interaction with TβRI, our study delves into understanding the impact of inhibiting these interactions on downstream signaling of both Integrins and TβRI, and their role in TNBC tumor progression and metastasis. Our findings confirm direct interactions between Kindlin-2, β1-Integrin, and TβRI, with Kindlin-2 crucial for stabilizing the β1-Integrin: TβRI protein complexes. Loss of Kindlin-2 expression leads to degradation of both β1-Integrin and TβRI proteins, which can be rescued by re-expression of Kindlin-2. Importantly, loss of Kindlin-2 expression inhibits downstream signaling pathways of both β1-Integrin and TβRI. The biological significance of Kindlin-2-mediated stabilization is reflected in the inhibition of oncogenic behavior in TNBC tumors lacking Kindlin-2, β1-Integrin, or TβRI, both in vitro and in in vivo mouse models.

In summary, our findings unveil a novel role for Kindlin-2 in simultaneously regulating the oncogenic activities of both β1-Integrin and TβRI by stabilizing the β1-Integrin:Kindlin-2:TβRI complex; an insight that holds promise for advancing our understanding and potential therapeutic interventions in TNBCs.

## Methods

### Cell lines and reagents

MDA-MB-231, 4T1, and HEK293 cells were procured from the American Type Culture Collection (ATCC; Manassas, VA) and maintained in accordance with the manufacturer’s specified protocols. Although cell line authentication was not explicitly conducted, we relied on the manufacturer’s quality control assurances. Periodic testing for Mycoplasma contamination was performed every 9 to 12 months. All cells were cultured at early passages (no more than 15), and each culture was passaged no more than five times before introducing a fresh vial. Kindlin-2, TβRI, and ITGB1-deficient cells were generated through electroporation of cancer cells with a ribonucleoprotein mixture of guide RNAs (sgRNA) and Cas9 (Synthego), following the manufacturer’s instructions (Sup. Table 1). A pool of three verified sgRNAs was used for each human or mouse gene (Synthego), with scrambled sgRNAs serving as a negative control. Western blot (WB) analysis validated efficient and stable knockout (KO). In cases where knockdown efficiency was below 80%, a second round of sgRNA delivery was implemented. No antibiotic selection was required, as the knockdown efficiency was sustained throughout the cells’ utilization. Primary antibodies for TβRI, TβRII, phospho-TβRI, phospho-TβRII, phospho-Smad2/3, Smad3 were obtained from Abcam, Inc. Mouse monoclonal anti-Kindlin-2, clone 3A3 was sourced from EMD Millipore. Mouse monoclonal anti-Kindlin-2 (EMD Millipore). PE-conjugated anti-Integrin β1/CD29 (GeneTex), FITC-conjugated HUTS-4 antibody (Sigma). Rabbit polyclonal anti-FAK (Invitrogen), Mouse monoclonal anti-Vinculin (Sigma). Secondary antibodies for IF were donkey anti-mouse IgG Alexa 594 and Donkey anti-Rabbit IgG Alexa 488 (Invitrogen). Goat horseradish peroxidase-conjugated anti-mouse IgG and goat horseradish peroxidase-conjugated anti-rabbit IgG for western blot (Bio-Rad). Gel electrophoresis reagents for protein and DNA were from Bio-Rad.

### Co-Immunoprecipitation and western blotting

Cells were lysed with RIPA or NP40 lysis buffer with proteases and phosphatases inhibitor cocktails. Total protein quantification was performed using the BCA protein assay kit (Bio-Rad). Co-immunoprecipitation analysis involved incubating lysates at 4°C with protein A resin and specific antibodies, as described previously [30]. Western blot assays followed standard protocols with β-Actin as the loading standard. The ChemiDoc MP Imaging system (Bio-Rad) was employed for image acquisition of developed membranes.

### Colony formation assay

MDA-MB-231 (3000 cells) and 4T1 (1000 cells) were seeded into 6-well plates. Cultured for 10 days, fresh medium was supplemented every 3 days. Clones were washed with PBS, fixed with 4% paraformaldehyde (PFA) at room temperature for 20 minutes, and stained with 0.25% crystal violet solution. Image acquisition of the 6-well plate and quantification of clones were performed using the ChemiDoc MP Imaging system (Bio-Rad) and ImageJ software.

### Wound healing assay

Cells were seeded in 6-well plates, grown to a confluent monolayer, and subjected to a scratch wound. After a quick wash with PBS, cells were cultured for 22 hrs. Images at 0 and 22 hrs. post-wounding were acquired using a Nikon ECLIPSE TS2r microscope, and the remaining open area was calculated using ImageJ software.

### RNA Extraction and quantitative real time RT- PCR

Total RNA was isolated using TRIzol reagent (Invitrogen) and quantified with Nanodrop. Reverse transcription and quantitative real-time PCR (Bio-Rad) were performed using the High Capacity cDNA Reverse Transcription Kit (Invitrogen) and SYBR Green Master Mix Kit (Invitrogen), respectively. Primers were obtained from Qiagen (Sup. Table 2).

### 3D-tumorsphere and invasion assays

For 3D single-tumorsphere formation, cells were seeded into a 96-well ultralow attachment (ULA) plate and monitored for 12 days, as described previously [31]. Invasion assays involved supplementing tumorspheres cultures with Matrigel, and invasion was monitored for 10 additional days. Images were captured and quantified using ImageJ software[31].

### Cell adhesion and spreading assays

Adhesion and spreading assays were performed as described previously [31]. Cells were seeded onto coverslips precoated with fibronectin, laminin, and Matrigel. Adhered cells were imaged, and spreading was assessed by capturing different fields and quantifying the area around the cells using ImageJ.

### Flow Cytometry analyses

Cell surface expression and activation of β1-Integrin was assessed by FACS (Sony ID7000), as described previously [31]. Cells were detached using trypsin, washed, and resuspended for staining with PE-conjugated mouse anti-human CD-29 antibody or FITC-conjugated HUTS-4 antibody. Data were analyzed using FlowJo software.

### Immunofluorescence and confocal microscopy

Cells were seeded on glass coverslips precoated with poly-L-Lysine, processed, and incubated with primary antibodies overnight. After blocking and incubating with secondary antibodies, cells were mounted with DAPI-containing mounting medium. Images were captured on a LEICA DM5500 laser scanning confocal microscope.

### In vivo tumor growth and metastasis study

NSG female mice and BALB/C mice were purchased from Jackson and used for tumor growth and metastasis studies as described in our published studies[32–35]. Parental and derivative cells were injected into mammary fat pads, and tumor growth was monitored. For lung metastasis assays, cells were injected into the tail vein. Mice were sacrificed, and tumors or metastases were analyzed [32–36].

### Statistical analysis

Statistical analyses were performed using GraphPad Prism (version 8.0) and SPSS (version 21.0). All experiments were conducted in triplicate, and variables were expressed as mean ± SD. Student’s t-test was used, and significance was considered at p < 0.05.

## Results

### Kindlin-2 is overactivated in triple negative breast cancer tumors

**Figure 1:**
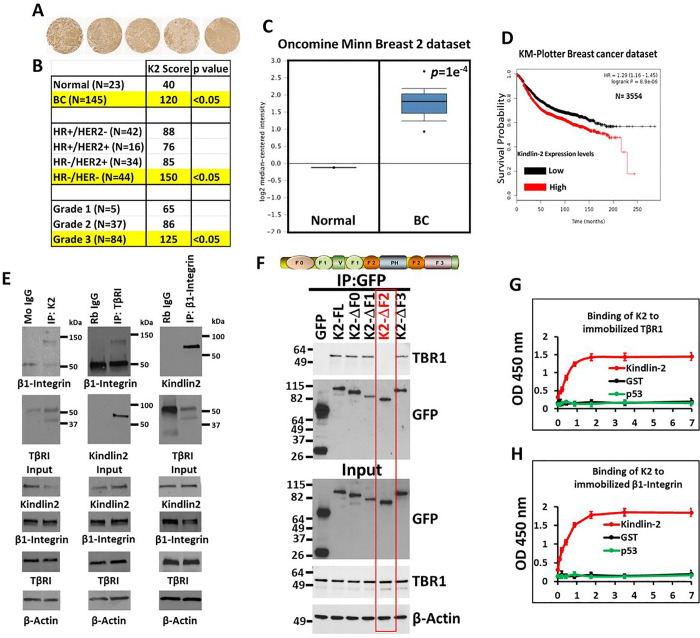
Kindlin-2 interacts with both β1-Integrin and TβRI: (A) Representative K2 immunostainings of advanced human BC tumors from a 150 BC TMA cohort. (B) Kindlin-2 IHC staining scores from panel B. (C) Oncomine datasets comparing K2 mRNA levels of normal and BC tissues. (D) KM plot correlating survival of 3954 BC patients with K2 expression levels. (E) Co-immunoprecipitation of total protein lysates from MDA-MB-MB-231 cells showing interactions between endogenous K2 β1-Integrin and TβRI. (F) Pull down assays showing that the F2 domain of K2 is necessary for the interaction between K2 and TβRI. (G&H) Solid binding assay monitoring the binding of recombinant Kindlin-2 to immobilized TβRI (G)or β1-Integrin (H).

Prior studies have established the role of Kindlin-2 as a major driver of tumor progression and metastasis in several cancers including the one that originates in the breast [15]. Published studies form our group and others have shown that Kindlin-2 is involved in the activation of the oncogenic behavior of BC tumors, both in vitro and in vivo [16, 17, 20, 22–25, 27, 28, 34]. Interrogation of a BC tumor microarray generated from BC specimens representing the different BC subtypes [16, 37], showed high levels Kindlin-2 staining in advanced BC stages (Fig. 1A&B). Kindlin-2 staining score was significantly (p < 0.05) higher in BC tumors compared to normal breast tissues (Fig. 1B). More importantly, Kindlin-2 staining score was significantly (p < 0.05) higher in tumor of basal (hormone receptor-negative and Her2-negative) subtype (Fig. 1B), a tread that was also found in human and murine cell lines of basal and triple negative BC (TNBC) nature [16, 37]. These findings were further confirmed by interrogation of public BC datasets from Oncomine (www.oncomine.org), where we found Kindlin-2 mRNA expression levels to be significantly (p < 0.001) higher in BC tumors compared to normal breast tissues (Fig- 1C). Furthermore, interrogation of the KM-Plotter BC database (https://kmplot.com/analysis/) showed increased expression levels of Kindlin-2 correlate with poor disease outcome in patients with BC tumors (Fig. 1D). These findings, together with our published studies [16, 17, 22–24, 34]) support the key role that Kindlin-2 plays in the pathology of BC tumors, in general, and in TNBC tumors, in particular. One of the major functions of Kindlin-2 is the activation of the inside-out signaling of integrins through its physical interaction with the cytoplasmic tail of several integrin β-subunits, including β1-Integrin (Reviewed in [26, 38]]). Previously [16], we showed that Kindlin-2 activates the CSF1/EGF paracrine oncogenic loop in TNBC through the regulation of TGF-β signaling. Interestingly, a study by Wei et al. [29] showed that Kindlin-2 also interacts with the cytoplasmic region of TGF-β type one receptor (TβRI). Therefore, our published studies have shown that Kindlin-2 plays a major role in the regulation of TNBC tumor progression and metastasis through the regulation of the oncogenic activities of both Integrins and TGF-β. Based on this information we sought to investigate the molecular mechanisms that regulate the Kindlin-2 interaction with both β1-Integrin and TβRI, and the role of these interactions in the regulation TNBC tumor progression and metastasis.

### Kindlin-2 interacts with both β1-Integrin and TβRI

Co-immunoprecipitation using total protein lysates from MDA-MB-213 TNBC cells showed Kindlin-2 immunocomplexes readily captured both TβRI and β1-Integrin (Fig. 1E, left panels), while TβRI immunocomplexes also readily captured both Kindlin-2 and β1-Integrin (Fig. 1E, middle panels), and β1-Integrin immunocomplexes readily captured Kindlin-2 and TβRI (Fig. 1E, right panels), thereby implicating Kindlin-2 as a potential adapter that coordinates the formation of TβRI and β1-Integrin complexes. In addition, pulldown assays identified the F2 domain within Kindlin-2 as being necessary for the interaction between Kindlin-2 and TβRI (Fig. 1F). The interaction between Kindlin-2 and cytoplasmic tail of β1-Integrin has already been established to be mediated via the Q^614^W^615^ amino acid doublet that resides within the F3 domain of Kindlin-2. Finally, surface plasmon resonance analyses (solid binding assay) monitoring the binding of recombinant Kindlin-2 to immobilized TβRI (Fig. 1G) or β1-Integrin (Fig. 1H), established a direct binding of Kindlin-2 to either TβRI and β1-Integrin, thereby establishing a physical bridge between TβRI and β1-Integrin.

### Kindlin-2 is required for the stabilization of the β1-Integrin:Kindlin-1:TβRI protein complex

**Figure 2:**
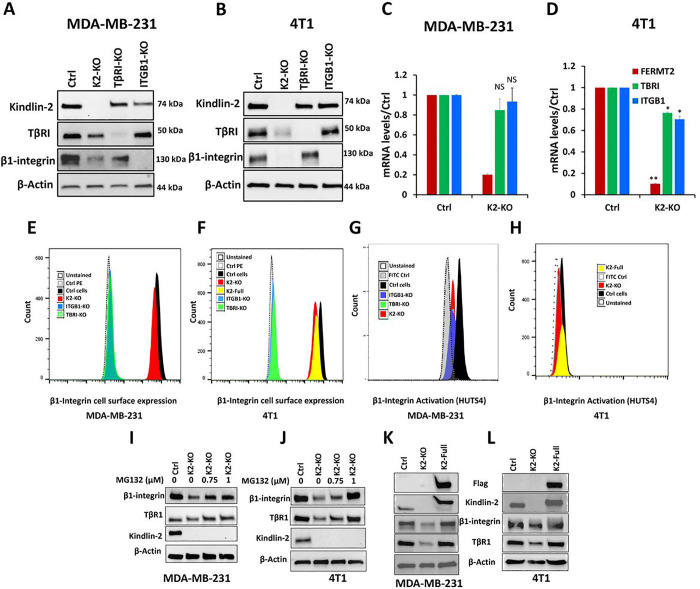
Kindlin-2 is required for the stabilization of the β1-Integrin:Kindlin-1:TβRI protein complex: MDA-MB-231 cells (A) and 4T1 cells (B) were subjected to CRISPR/Cas 9 mediated gene editing to knockout expression of FERMT 2 (K2) ITGB 1 (β1 Integrin) or TβRI genes, and their protein expression was assessed by Western Blot analysis. Β-Actin is a loading control. mRNA expression levels of TβRI and β1-Integrin in the K2-KO MDA-MB-231 (C) and 4T1 (D) TNBC cells. (E,F,G,H) Flow cytometry diagrams of the cell surface expression levels of β1-Integetrin (E&F) and its activated form (G&H) in K2-KO, TβRI-KO, ITGB1-KO and K2-full rescued K2-KO MDA-MB-231 cells. (I,J,K,L) Expressions of TβRI and β1-Integrin proteins in MDA-MB-231 K2-KO after treatment with MG132 (I&J) or after rescue of K2 full length (K&L). Data are the mean ± SD (n = 3, *p < 0.05, Student’s t test).

Probing further into the importance of Kindlin-2 in maintaining the integrity of TβRI/Kindlin-2/β1-Integrin protein complexes, we found loss of expression of Kindlin-2 (K2-KO) in MDA-MB-231 (Fig. 2A) or 4T1 (Fig. 2B) TNBC cells leads to the degradation of both TβRI and β1-Integrin proteins. mRNA expression levels of either TβRI or β1-Integrin were not significantly affected in the K2-KO MDA-MB-231 (Fig. 2C) or 4T1 (Fig. 2D) TNBC cells, suggesting that loss of expression of TβRI and β1-Integrin in K2-KO cells was a result of protein degradation, but not at the mRNA transcription levels. We also used flow cytometry to measure the cell surface expression levels of β1-Integetrin and found loss of expression (KO) of either Kindlin-2, TBRI or β1-Integrin resulted in inhibition of expression of β1-Integrin expression at the surface of the cell membrane where it exerts it’s signaling functions (Fig. 2E). Over-expression of full-length Kindlin-2 (K2-full) in the K2-KO MDA-MB-231 cells restored cell surface expression of β1-Integrin to levels comparable to those found in the control cells (Fig. 2F). We also used the HUTS4 assay that measures the active state of β1-Integrin [39], and found loss of expression of either Kindlin-2, TβRI or β1-Integrin resulted in inhibition of activation levels of β1-Integrin (Fig. 2G), which could also be restored by over-expressing of full-length Kindlin-2 (K2-full) in the K2-KO cells (Fig. 2H). Moreover, treating the K2-KO MDA-MB-231 (Fig. 2I) or 4T1 (2J) cells with the proteasome inhibitor MG132 resulted in the restoration of both TβRI and β1-Integrin proteins to levels found in the control cells, meanwhile over-expression of full-length Kindlin-2 in the K2-KO restored protein expression of both TβRI or β1-Integrin (Fig. KI & 2L). Therefore, these data support the role of Kindlin-2 in maintaining the physical integrity of the TβRI:Kindlin-2:β1-Integrin protein complex.

### Loss of expression of either Kindlin-2, TβR1 or β1-Integrin inhibits the in vitro oncogenic behavior of TNBC tumors, and re-expression of Kindlin-2 is sufficient for the restoration of these oncogenic activities

**Figure 3:**
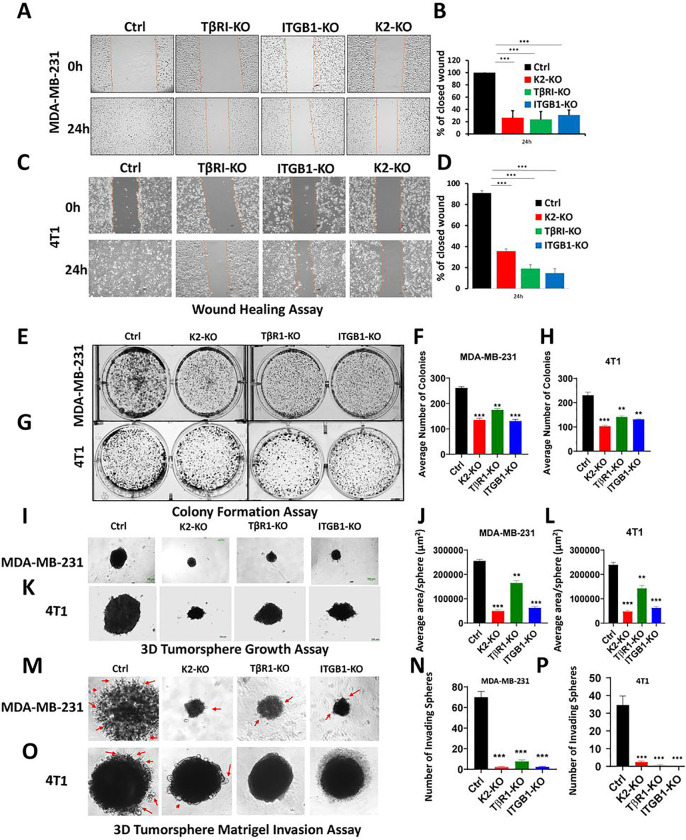
Loss of expression of either Kindlin-2, TβR1 or β1-Integrin inhibits the in vitro oncogenic behavior of TNBC tumors: Wound healing assay (A,B,C,D), 2D-colony formation assay (E,F,G,H), 3D-tumorsphere growth assay (I,J,K,L), and 3D-tumorsphere invasion assay (M,N,O,P) of MDA-MB-231 and 4T1 cells (Ctrl, K2-KO, TβR1-KO, ITGB1-KO). Data are the mean ± SD (n = 3, **p < 0.01; ***p < 0.001, Student’s t test).

**Figure 4:**
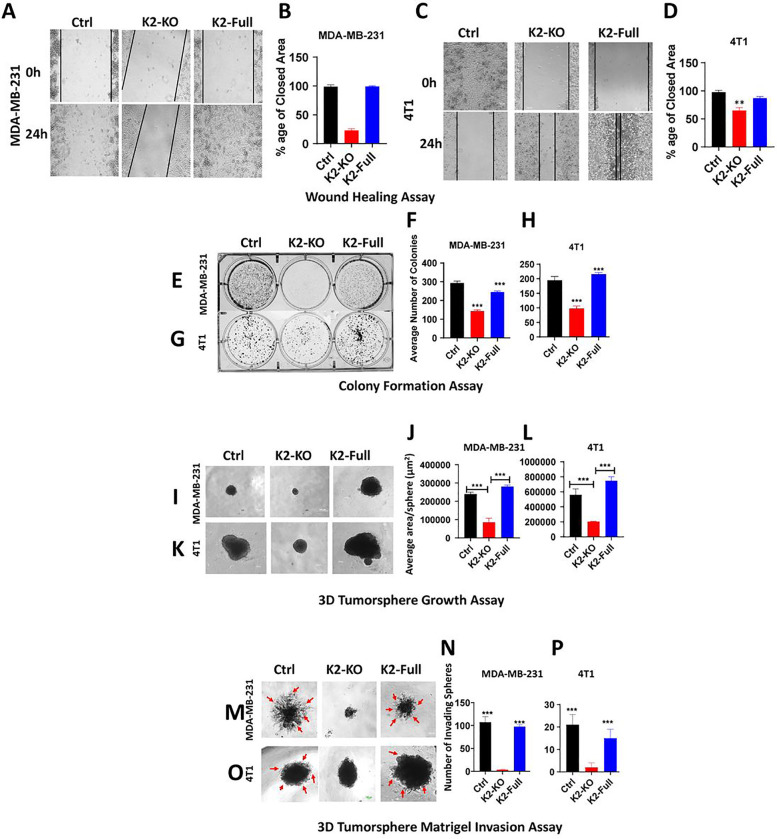
Re-expression of Kindlin-2 expression rescues the in vitro oncogenic behavior of TNBC tumors: Wound healing assay (A,B,C,D), 2D-colony formation assay (E,F,G,H), 3D-tumorsphere growth assay (I,J,K,L), and 3D-tumorsphere invasion assay (M,N,O,P) of MDA-MB-231 and 4T1 cells (Ctrl, K2-KO, rescued K2-full). Data are the mean ± SD (n = 3, **p < 0.01; ***p < 0.001, Student’s t test).

To investigate the biological significance of loss of expression of either Kindlin-2, TβR1 or β1-Integrin (ITGB1), and their effect on the oncogenic behavior of TNBC cells, we performed several *in vitro* assays. Parental MDA-MB-231 or 4T1, or their Kindlin-2-, TβR1- or ITGB1-deficient (KO) derivatives were subjected to the wound healing assay (Fig. 3A–D). Loss of expression of either Kindlin-2, TβR1 or ITGB1 resulted in the inability of the MDA-MB-231 KO cells (Fig. 3A&B) or the 4T1 KO cells (Fig. 3C&D) to effectively close the scratch wound after 24 h, supporting the role of Kindlin-2, TβR1 and β1-Integrin in cancer cell migration. Loss of expression of either Kindlin-2, TβR1 or ITGB1 also inhibited the colony formation potential, a hallmark of cancer cells phenotype, of both MDA-MB-231 (Fig. 3E&F) and 4T1 (Fig. G&H) cells. To mimic the behavior of cancer cells in tumor microenvironment, we performed 3D-tumorsphere growth and tumorsphere invasion of extracellular matrices (ECMs). 3D-tumorsphere growth was significantly (p < 0.01) inhibited in both the MDA-MB-231 (Fig. I&J and Sup Fig. 1A) and the 4T1 (3K&L and Sup. Figure 1B) cells deficient in either Kindlin-2, β1-Integrin or ITGB1 K2-KO. Similarly, MDA-MB-231 or 4T1 cells (Fig. 3M&N and Fig. 3O&P, respectively) were unable to invade ECM containing Matrigel (Fig. 3I–P, and Sup. Figure 2A&B). Interestingly re-expression of full-length Kindlin-2 (K2-Full) in K2-KO MDA-MB-231 or 4T1 cells restored their cell migration potential of MDA-MB-231 cells (Fig. 4A&B) and 4T1 cells (Fig. 4C&D). Colony formation activity was also restored in the K2-KO MDA-MB-231 and 4T1 cells re-expressing K2-Full (Fig. EG&F and Fig. 4G&H, respectively). In a similar manner, the ability of MDA-MB-231 and 4T1 cells to establish tumorspheres in 3D organoid growth assays (Fig. 4I–L, and Sup Fig. 3A&B) and for these tumorspheres to invade ECMs (Fig. 4M–P, and Sup Fig. 4A&B) were also restored in the K2-KO MDA-MB-231 and 4T1 cells re-expressing full-length Kindlin-2. These data have so far demonstrated the requirement of Kindlin-2, TβR1 and ITGB1 for the common oncogenic activities of cancer cells, and for Kindlin-2 to be sufficient to restore these activities downstream of either β1-Integrin and TβRI.

### Loss of expression of either Kindlin-2, TβRI or ITGB1 inhibits signaling activities that specific to β1-Integrin and TβRI

**Figure 5:**
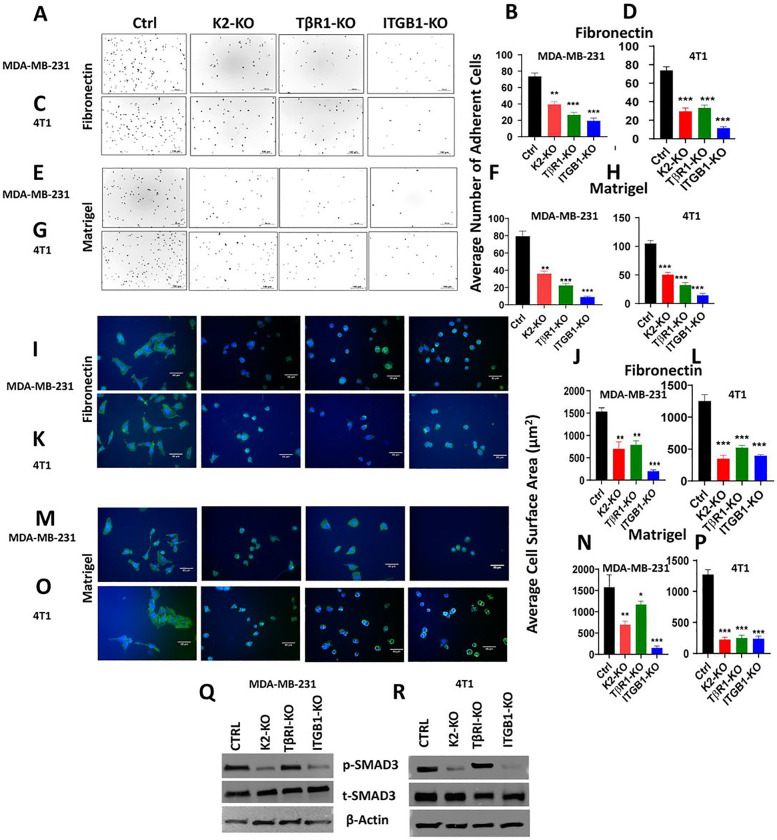
Loss of expression of either Kindlin-2, TβRI or ITGB1 inhibits signaling activities that specific to β1-Integrin and TβRI: Representative pictures and average number of adherent MDA-MB-231 and 4T1 cells (Ctrl, K2-KO, TβR1-KO, ITGB1-KO) on fibronectin (A,B,C,D), and on Matrigel (E,F,G,H), Pictures and average cell surface area of MDA-MB-231 and 4T1 cells (Ctrl, K2-KO, TβR1-KO, ITGB1-KO) on fibronectin (I,J,K,L), and on Matrigel (M,N,O,P) after spreading assay. (Q,R), WB results of phosphorylation levels of SMAD2/3 in both MDA-MB-231 and 4T1 cells (Ctrl, K2-KO, TβR1-KO, ITGB1-KO). Data are the mean ± SD (n = 3, **p < 0.01; ***p < 0.001, Student’s t test).

In the next set of experiments, we investigated the effect of loss of either Kindlin-2, TβR1 or ITGB1 on oncogenic activities that are specifically activated downstream of either β1-Integrin or TβRI. For the β1-Integrin downstream activities, we assessed for cell adhesion on fibronectin, a cellular activity that regulated by integrins [40] (Fig. 5A–H). Loss of expression of either of the three genes resulted in a significant (p < 0.001) inhibition of cell adhesion of MDA-MB-231 and 4T1 cells to Fibronectin (Fig. 5A&B and Fig. 5C&D, respectively). Adhesion of MDA-MB-231 and 4T1 cells to Matrigel was also significantly (p < 0.001) inhibited because of loss of expression of either of the three proteins (Fig. 5E&F and Fig. 5G&H, respectively). Cell adhesion to Laminin was also affected as a result of loss of expression of either of three genes (Sup. Figure 5). Cell spreading on ECM is also a cellular activity that is tightly regulated by integrins [40]. Here again, we found loss of expression of either Kindlin-2, TβR1 or ITGB1 resulted in a significant (p < 0.001) inhibition of spreading of MDA-MB-231 and 4T1 to fibronectin (Fig. 5I&J and Fig. 5K&L, respectively) to Matrigel (Fig. 5M&N and Fig. O&P, respectively), or to Laminin (Sup. Figure 6). Thus we show loss of expression of either Kindlin-2, TβR1 or ITGB1 to inhibits cellular activities that are specific to Integrins. Since β1-Integrin can form heterodimers with other α-Integrin subunits [41][42], we used qt-RT-PC to assess for expression levels of α1, αV and α5, which are predominantly expressed in cancer cells [43]. Loss of Kindlin-2 did not affect expression levels of either α1, αV and α5 subunits (Sup. Figure 7A). However, loss of either ITGB1 or TβR1 resulted in a significant (p < 0.01) inhibition of expression of both αV and α5 subunits, but those of α1 subunit (Sup. Figure 7B and 7C, respectively). This is consistent with the spreading data obtained on Fibronectin and Lamin since both αV and α5 integrin subunits, but not α1 subunit are involved in the β1-Integrin-mediated interaction with Fibronectin and Laminin [42]. Finally, as a readout for the molecular signaling downstream of TβRI, we assessed for phosphorylation levels of SMAD2/3 that takes place downstream of the TGF-β-mediated activation of the TβRI:TβRII complex. Loss of expression of either of the three proteins also resulted in reduction in phosphorylation levels of SMAD2/3 in both MDA-MB-231 (Fig. 5Q) and 4T1 cells (Fig. 5R). Levels of basal pSMAD2/3 did not increase in the KO cells even after stimulation of TGF-β (Sup. Figure 8A). Phosphorylation levels of TβRII, the upstream effector of SMAD, were also inhibited in the KO cells (Sup. Figure 8B). Thus, we show that loss of expression of either Kindlin-2, TβR1 or ITGB1 inhibited the TβRI-specific downstream signaling, and, therefore, implicating Kindlin-2 as a major player in the regulation of the downstream signaling effectors of both β1-Integrin and TβR1.

### Re-expression of Kindlin-2 in the K2-deficient TNBC cells is sufficient for the restoration of the oncogenic activities downstream of β1-Integrin and TBRI

**Figure 6:**
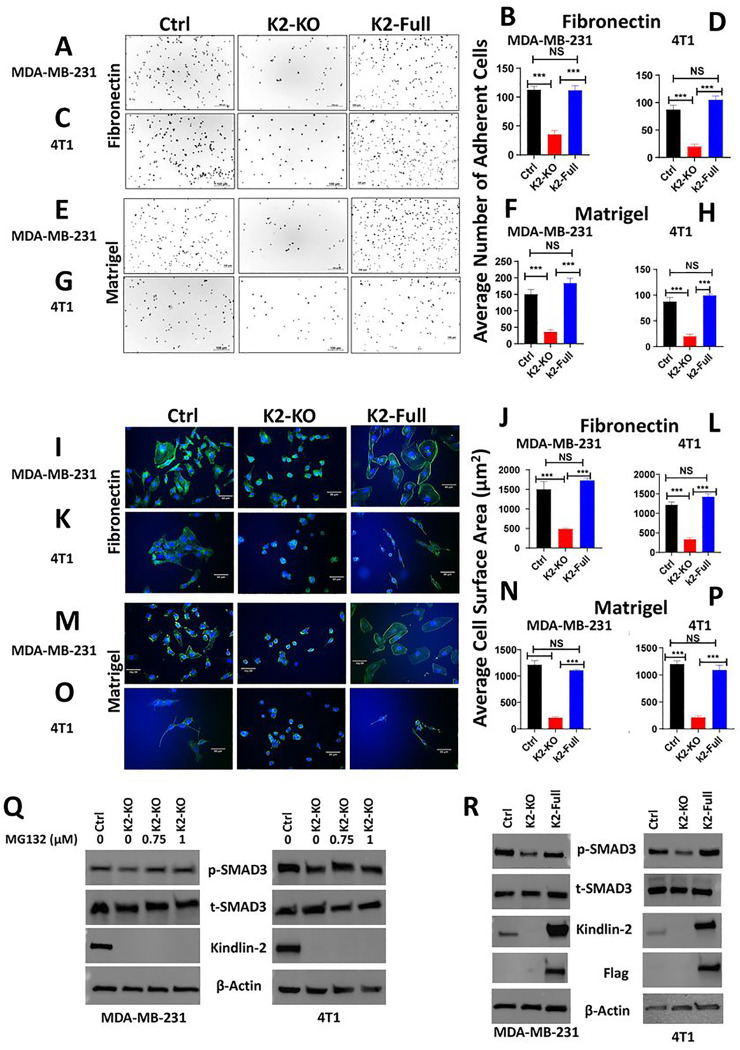
Re-expression of Kindlin-2 in the K2-deficient TNBC cells is sufficient for the restoration of the oncogenic activities downstream of β1-Integrin and TBRI: Representative pictures and average number of adherent MDA-MB-231 and 4T1 cells (Ctrl, K2-KO, rescued K2-full) on Fibronectin (A,B,C,D), and on Matrigel (E,F,G,H), Pictures and average cell surface area on MDA-MB-231 and 4T1 cells (Ctrl, K2-KO, rescued K2-full) after spreading assay on fibronectin (I,J,K,L) and on Matrigel (M,N,O,P). (Q,R), WB results of phosphorylation od SMAD3 in MDA-MB-231 K2-KO after treatment with MG132 and in K2-KO cells re-expressing full length K2. Data are the mean ± SD (n = 3, ***p < 0.001, Student’s t test).

To determine whether the cellular activities that are regulated downstream of β1-Integrin are mediated by Kindlin-2, the K2-KO MDA-MB-231 and 4T1 TNBC cells re-expressing full-length Kindlin-2 were subjected to cell adhesion and spreading. Cell adhesion on fibronectin was fully restored in both K2-deficient MDA-MB-231 cells (Fig. 6A&B) and 4T1 cells (Fig. 6C&D) re-expressing Kindlin-2. Cell adhesion on Matrigel was also fully restored for both cell lines (Fig. 6E&F, and Fig. 6G&H), as well as Laminin (Sup. Figure 9). Similarly, re-expression of full length Kindlin-2 in the K2-KO cells also fully restored the spreading potential of both cell lines on both fibronectin (Fig. 6I&J and Fig. 6K&L for MDA-MB-231 and 4T1, respectively), Matrigel (Fig. 6M&N and Fig. 6O&P for MDA-MB-231 and 4T1), and Laminin (Sup. Figure 10). Phosphorylation levels of SMAD, a readout of TβRI-specific downstream signaling activity, was also restored in K2-KO MDA-MB-231 cells treated with the proteosome inhibitor MG132 (Fig. 6Q), as well as in the K2-KO cells re-expressing full length Kindlin-2 (Fig. 6R). Thus we confirm that the signaling activities that are mediated downstream of β1-Integrin or TBRI are specifically regulated by Kindlin-2. additionally, we confirmed that Kindlin-2 expression, by stabilizing the β1-Integrin:TβRI protein complex is sufficient for the restoration of these β1-Integrin and TβRI downstream cellular and signaling activities.

### Loss of expression of either Kindlin-2, TβRI or ITGB1 inhibits growth and metastasis of TNBC tumors, which can be restored buy re-expression of Kindlin-2

**Figure 7:**
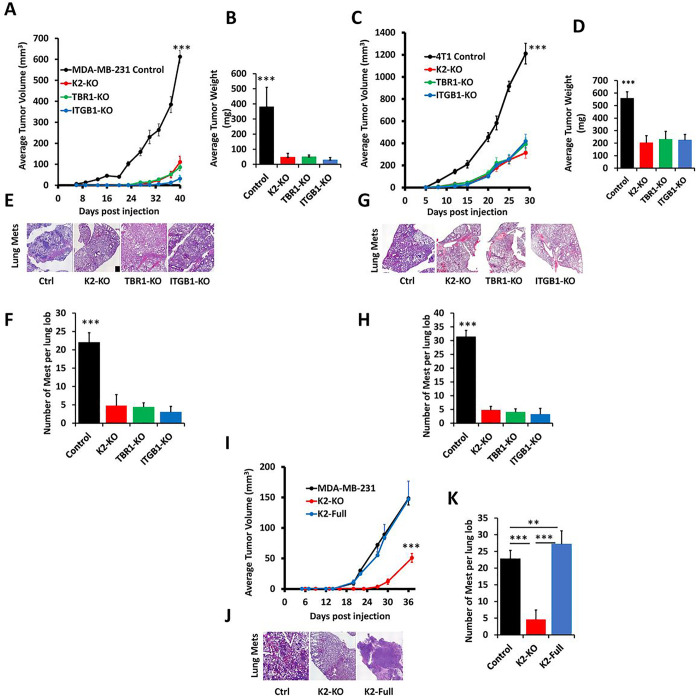
Loss of expression of either Kindlin-2, TβRI or ITGB1 inhibits growth and metastasis of TNBC tumors, which can be restored buy re-expression of Kindlin-2: Tumor volume and weight in both the MDA-MB-231 NSG mice and 4T1 Balb/C mice models up to 40 days post injection (A,B,C,D), and representative H&E staining of their corresponding lungs showing metastasis foci. (E,F,G,H), Tumor volume in the MDA-MB-231 NSG mice model up to 36 days post injection with re-expression of K2 in the MDA-MB-231 K2-KO cells (I), and representative H&E staining of their corresponding lungs showing metastasis foci (J,K).

Next, we determined whether the biological and signaling effects observed in vitro, can also be recapitulated in vivo in mouse models for TNBC tumor progression and metastasis. Using the spontaneous metastasis mouse model, MDA-MB-231 or 4T1 TNBC control cells and their KO derivatives were injected in the mammary fat pads of NSG mice (MDA-MB-231) or Balb/C mice (4T1), and growth of the primary tumors was monitored over time. Loss of expression of either of the three proteins resulted in a significant (p < 0.001) delay in tumor growth and weight in both the MDA-MB-231 model (Fig. 7A&B) as well as the 4T1 model (Fig. 7C&D). Metastasis was also significantly (p < 0.001) inhibited in the lungs of mice injected with the KO MDA-MB-231 cells (Fig. 7E&F) or with KO 4T1 cells (Fig. G&H). Re-expression of Kindlin-2 in the Kindlin-2-deficient MDA-MB-231 cells resulted in the rescue of both tumor growth (Fig. 7I) and metastasis (Fig. 7J&K). These data show the impact of Kindlin-2, TβRI and ITGB1on tumor progression and metastasis. They also confirm the specificity of Kindlin-2 in the process, where Kindlin-2 is sufficient for the restoration of the growth and metastasis potentials of TNBC tumors that lack expression of either TβRI or ITGB1.

## Discussion

Kindlin-2 initially garnered attention for its pivotal role in activating integrins, thereby mediating cell-extracellular matrix adhesion and signaling [24, 44–49]. This function is crucial for facilitating the interaction between cells and their extracellular environment by modulating integrin activity [24, 44–49]. Integrins, as transmembrane receptors, play a key role in cell adhesion and signal transduction. Kindlin-2 binds to the cytoplasmic tails of integrins, promoting their activation and enabling interaction with extracellular ligands. This activation is vital for cell adhesion, migration, and communication with the surrounding microenvironment. The dynamic interplay between Kindlin-2 and integrins significantly contributes to cell behavior under both normal physiological conditions and pathological manifestations such as cancer. In normal cellular functions, this relationship is crucial for embryonic development, tissue homeostasis, and immune responses [15]. However, dysregulation of Kindlin-2 and integrin interactions has been linked to various diseases, particularly cancer, where aberrant cell adhesion and migration are prominent features [13].

Beyond its role in integrin activation, Kindlin-2 has emerged as a key player in regulating transforming growth factor-beta (TGF-β) signaling, the activating ligand of the TβRI:TβRII signaling complex [29, 50]. This interplay between Kindlin-2 and TGF-β regulates various cellular processes and contributes to both normal development and pathological conditions, including breast cancer (BC) pathology [16]. The Kindlin-2-TGF-β axis plays a crucial role in epithelial-mesenchymal transition (EMT), a process central to embryonic development and implicated in cancer metastasis [17, 51]. Dysregulation of TGF-β signaling is a hallmark of cancer progression, and Kindlin-2 has been implicated in mediating TGF-β effects on tumor cell behavior [16, 17]. Notably, the interaction between Kindlin-2 and TGF-β receptors enhances cellular responsiveness to TGF-β and its downstream signaling, influencing processes such as cell proliferation and differentiation [29, 52]. In turn, TGF-β signaling also regulates Kindlin-2 expression.

Previously, our studies demonstrated that Kindlin-2 activates the CSF1/EGF paracrine oncogenic loop in BC through the regulation of TGF-β signaling [16]. Additionally, a study by Wei et al. [29] revealed Kindlin-2’s binding to the cytoplasmic region of the TGF-β type one receptor (TβRI). Consequently, Kindlin-2 plays a major role in regulating triple negative breast cancer (TNBC) tumor progression and metastasis through the modulation of the oncogenic activities of both integrins and TGF-β.

In this paper, we present, for the first time to the best of our knowledge, the direct binding of Kindlin-2 to TβRI and β1-Integrin, establishing a physical bridge between TβRI and β1-Integrin. Moreover, Kindlin-2 not only is necessary for the stabilization of the TβRI:Kindlin-2:β1-Integrin complex but is also required to maintain the oncogenic behavior of TNBC cell lines both in vitro and in vivo. Indeed, Kindlin-2 is a crucial protein involved in integrin-mediated adhesion and signaling processes, essential for cell-extracellular matrix adhesion [53]. Kindlin-2 is believed to associate with β1-Integrin at nascent adhesions before talin recruitment during adhesion maturation, indicating its early involvement in the adhesion process [54] (Sup. Figures 11 and 12). Disrupting the interaction between the Kindlin-2 dimer inhibits adhesion formation, integrin activation, and cell spreading, underscoring the significance of Kindlin-2 in these processes [38, 55–57].

Conversely, TβRI plays a vital role in the TGF-β/SMAD signaling pathway, regulating cell growth, differentiation, and migration, making it a central mediator of cancer progression [58]. Studies on TβR1 and SMAD in breast cancer emphasize the multifaceted role of the TGF-β signaling pathway and its components in the disease. While genetic variants such as TβRI*6A have shown associations with breast cancer risk and progression, the regulatory mechanisms and functional implications of TβR1 and SMAD signaling in breast cancer require further investigation for a comprehensive understanding of their potential as therapeutic targets. Our study demonstrates that the loss of expression of Kindlin-2, TβR1, or β1-Integrin proteins results in a reduction in phosphorylation levels of SMAD2/3 in both MDA-MB-231 and 4T1 cells. Activated TβR1 phosphorylates SMAD2 and SMAD3, which heterodimerize with SMAD4 and translocate to the nucleus, binding to DNA and regulating the transcription of target genes involved in various cellular functions [59]. A recent study has also reported on the interplay between β1-Integrin, Kindlin-2, and TBRI partner, TBRII, to promote pancreatic tumor growth [52]. This study establishes the molecular mechanism by which the interplay between these oncogenic proteins is regulated in the context of breast cancer progression and metastasis.

Our investigation employed a combination of in vitro assays, two different mouse models for TNBC tumors, as well as genetic and pharmacological manipulation to establish Kindlin-2’s role as a major contributor to the stabilization of the TβRI:β1-Integrin protein complexes and the regulation of their downstream oncogenic activities, driving the progression and metastasis of TNBC tumors. We show that Kindlin-2 establishes direct interactions with TβRI and β1-Integrin, with the interaction between Kindlin-2 and TβRI mediated through the F2 domain of Kindlin-2 and the interaction between Kindlin-2 and β1-Integrin mediated through the F3 domain of Kindlin-2 via a QW amino acid doublet. CRISPR/Cas9-mediated knockout of Kindlin-2 leads to the loss of both TβRI and β1-Integrin proteins, resulting in the destabilization of the protein complex, which can be rescued by inhibiting the proteasome degradation machinery or re-expressing full-length Kindlin-2 in Kindlin-2-deficient cells. This supports the novel function of Kindlin-2 in establishing a physical bridge between TβRI and β1-Integrin and the requirement of Kindlin-2 for the stabilization of this complex. Loss of expression of either Kindlin-2, TβRI, or β1-Integrin leads to the inhibition of in vitro and in vivo oncogenic behavior of TNBC cells, highlighting the importance of either member of this protein complex in maintaining the oncogenic behavior of cancer cells; loss of one member of this complex is sufficient for the mitigation of the oncogenic activities of cancer cells. The downstream signaling effectors specific to either TβRI (phosphorylation of SMAD2/3) or β1-Integrin (cell adhesion and spreading on fibronectin) can be inhibited by simply losing Kindlin-2, emphasizing the specificity of Kindlin-2 in modulating TβRI- and β1-Integrin-mediated regulation of the oncogenic behavior of cancer cells.

**Figure 8:**
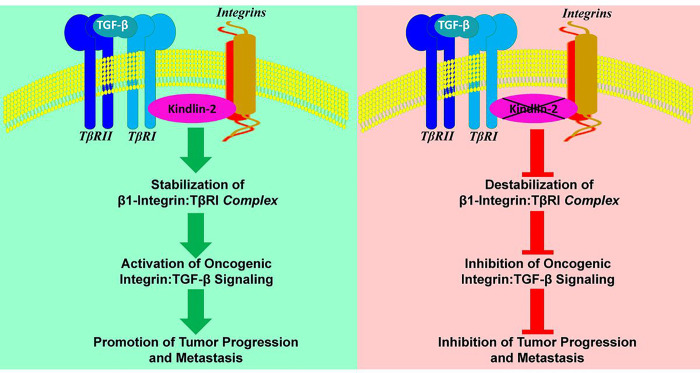
Model depicting the role of kindlin-2 as a physical bridge maintaining the stability of the β1-Integrin:K2:TβRI protein complexes and their effect in the regulation of the downstream oncogenic behavior of TNBC tumors. Data are the mean ± SD (n = 5 mice, **p < 0.01; ***p < 0.001, Student’s t test).

Our study unveils, for the first time, the intricate relationship between Kindlin-2, integrins, and TβRI, regulating crucial cellular processes (Fig. 8). This trilateral interplay integrates the roles of focal adhesion protein Kindlin-2, transmembrane receptors integrins, and TβRI in orchestrating various physiological and pathological events. At the core of this relationship lies the modulation of integrin activation by Kindlin-2. Kindlin-2 interacts with the cytoplasmic tails of integrins, promoting their activation and facilitating the connection between cells and the extracellular matrix (ECM). This activation is essential for cellular adhesion and sets the stage for downstream signaling events. On the other hand, Kindlin-2 influences the TGF-β signaling pathway through its interaction with TβRI, enhancing its activation and promoting downstream TGF-β signaling cascades. This interaction contributes to the regulation of cellular processes such as proliferation, differentiation, and migration, emphasizing the central role of Kindlin-2 in integrating signals from both integrins and TGF-β. Importantly, in a clinical setting, dysregulation of this Kindlin-2:Integrins:TβRI axis has implications in diseases such as cancer, where aberrant integrin activation and TGF-β signaling are hallmarks of cancer progression, and Kindlin-2 emerges as a potential key player bridging these pathways.

## Conclusions

In conclusion, the β1-integrin:Kindlin-2:TβRI interconnection represents a sophisticated network that regulates BC progression and metastasis. Further investigations of this trilateral oncogenic axis holds promise for deciphering the intricacies of cellular behavior in disease, and therefore offering potential therapeutic avenues to specifically target these pathways for the treatment of TNBC tumors and other tumors.

## Data Availability

All data are contained within the article. Requests for reagents should be addressed to DC Altieri (kxs586@case.edu).

## Abbreviations

BC: Breast cancer
TNBC: Triple negative breast cancer
ER: estrogen receptor
PR: progesterone receptor
HER2: human epidermal growth factor receptor
FERMT2: Fermitin family homolog 2
TGF-β: Transforming growth factor beta
ITGB1: Integrin beta 1
TβRI: Transforming growth factor type 1 receptor
TβRII: Transforming growth factor type 2 receptor
CRISPR: clustered regularly interspaced short palindromic repeats
KO: Knockout
CSF1: Colony stimulating factor 1
EGF: epidermal growth factor
CoI: Co-immunoprecipitation
IF: Immunofluorescence
H&E: Hematoxylin and Eosin
EMT: Epithelial to mesenchymal transition
Ab: antibody
IHC: immunohistochemistry
sgRNA: small guide RNA
mRNA: Messenger RNA
qRT-PCR: quantitative reverse transcription polymerase chain reaction
KM Plotter: Kaplan Meier Plotter
EMC: Extracellular matrix
